# In vitro response of THP-1 derived macrophages to antimicrobially effective PHMB-coated Ti6Al4V alloy implant material with and without contamination with *S. epidermidis and P. aeruginosa*

**DOI:** 10.1186/s40824-021-00247-1

**Published:** 2022-01-09

**Authors:** Paula Zwicker, Thomas Schmidt, Melanie Hornschuh, Holger Lode, Axel Kramer, Gerald Müller

**Affiliations:** 1 Institute of Hygiene and Environmental Medicine, Ferdinand-Sauerbruch-Str., University Medicine, D-17475 Greifswald, Germany; 2 Department of Pediatric Hematology and Oncology, Ferdinand-Sauerbruch-Str., University Medicine, D-17475 Greifswald, Germany

**Keywords:** Poly (hexamethylene biguanide) hydrochloride (PHMB), THP-1 macrophages, Ti6Al4V alloy, Implant surface, Cytokine secretion

## Abstract

**Aim:**

Periprosthetic joint infections are a devastating complication after arthroplasty, leading to rejection of the prosthesis. The prevention of septic loosening may be possible by an antimicrobial coating of the implant surface. Poly (hexamethylene) biguanide hydrochloride [PHMB] seems to be a suitable antiseptic agent for this purpose since previous studies revealed a low cytotoxicity and a long-lasting microbicidal effect of Ti6Al4V alloy coated with PHMB. To preclude an excessive activation of the immune system, possible inflammatory effects on macrophages upon contact with PHMB-coated surfaces alone and after killing of *S. epidermidis* and *P. aeruginosa* are analyzed.

**Methods:**

THP-1 monocytes were differentiated to M0 macrophages by phorbol 12-myristate 13-acetate and seeded onto Ti6Al4V surfaces coated with various amounts of PHMB. Next to microscopic immunofluorescence analysis of labeled macrophages after adhesion on the coated surface, measurement of intracellular reactive oxygen species and analysis of cytokine secretion at different time points without and with previous bacterial contamination were conducted.

**Results:**

No influence on morphology of macrophages and only slight increases in iROS generation were detected. The cytokine secretion pattern depends on the surface treatment procedure and the amount of adsorbed PHMB. The PHMB coating resulted in a high reduction of viable bacteria, resulting in no significant differences in cytokine secretion as reaction to coated surfaces with and without bacterial burden.

**Conclusion:**

Ti6Al4V specimens after alkaline treatment followed by coating with 5–7 μg PHMB and specimens treated with H_2_O_2_ before PHMB-coating (4 μg) had the smallest influence on the macrophage phienotype and thus are considered as the surface with the best cytocompatibility to macrophages tested in the present study.

**Supplementary Information:**

The online version contains supplementary material available at 10.1186/s40824-021-00247-1.

## Introduction

The number of endoprosthetic interventions is rising, with periprosthetic infections as one of the most frequent complications [1]. The colonization predominantly with species of the resident skin flora such as *Staphylococcus (S.) aureus, S. epidermidis* and *Cutibacterium acnes* [2–4] can result in the formation of biofilms, followed by rejection of the prosthesis and a need for replacement in many cases [5, 6]. The inoculation of perioperative contamination during the surgery often results from microorganisms in the depth of the skin surrounding the surgical field [7].

A prevention of such infections is possible by surfaces, that repel bacteria due to their surface characteristics, as superhydrophobic surfaces [8, 9]. However, these surfaces would also repel human cells, impeding an ingrowth of the implant [10]. Thus, these surfaces may be useful for the application on implants that have to be removed after a certain time.

Another possibility is coating the implant material with antimicrobial agents [11]. Existing coatings use biocides that are released from the surface, as antibiotics, antimicrobial peptides [12], antiseptics or metal ions [13–16]. However, the release of antibiotics and some antiseptics as e.g. chlorhexidine [17] into body fluids can provoke the formation of tolerance or resistance [18–22]. Additionally, metal ions [20, 21] and most synthetic biocide agents are not only antimicrobially effective, but also harm human cells. As an alternative, non-leachable coatings that kill bacteria upon contact [23, 24] as for instance tightly adsorbed poly (hexamethylene) biguanide hydrochloride [PHMB] can minimize the probability of bacterial resistance formation. This even in presence of organic burden and blood still effective antiseptic agent with broad spectrum activity [25] was adsorbed onto Ti alloy surfaces by Müller et al. and Hornschuh et al. after treating the Ti6Al4V surface with either H_2_O_2_ or NaOH [26–28].

Until recently, PHMB’s efficacy was ascribed to its polycationic properties, selectively damaging the microbial plasma membrane comprising acid phospholipids, leading to leakage of cell organelles and K^+^, and finally resulting in cell death [29, 30]. The main compounds of the plasma membrane of Gram-positive as well as Gram-negative bacteria are anionic glycerophospholipids as phosphatidylglycerol and diphosphatidylglycerol, besides other phospholipids. In contrast, these compounds are not found in the cell membrane of humans or other mammals, that contain mainly phosphatidylcholine (lecithin) [31], explaining the high tolerance of eukaryotic cells to PHMB. However, the interaction of PHMB with anionic phospholipids of the plasma membrane seems not to be the only reason for its antimicrobial efficacy. Zaki et al. pointed out that the PHMB-polymer can assemble in a hairpin-like structure that collapses further into a “snail-line” conformation with intense stacking by the biguanide groups [32] resulting in nano-objects that can interact with the phospholipid membrane similar to positively charged nanoparticles or cell-penetrating peptides [33], intracellularly producing an antimicrobial effect [32]. Both, mammalian as well as bacterial cells absorb PHMB in this manner. However, just bacterial chromosomes are clogged while mammalian cells encapsulate the antimicrobial agent in endosomes instead [34]. Since no DNA-SOS repair system is activated and no genotoxic or epigenetic effects are documented to date, the intracellular antimicrobial effect of PHMB is not associated with DNA degradation. This selective chromosome condensation provides an unanticipated paradigm for antimicrobial action that may not succumb to resistance [35].

Müller et al. demonstrated the antimicrobial effects of PHMB adsorbed on Ti6Al4V surfaces after exposure to *S. aureus*, *S. epidermidis*, *E. coli, and P. aeruginosa* [26]. The cell adherence and proliferation of osteoblast-like MG63 cells were not affected negatively. However, an immunological response has to be excluded, because macrophages attaching to implant surfaces can recognize the implant as a foreign body, leading to the secretion of inflammatory cytokines. Since these cytokines provoke an acute inflammatory process [36–38], macrophages are another main cause for implant failure. Thus, in the present study, the impact of the PHMB-coated Ti6Al4V surfaces on monocyte-derived THP-1 macrophages was investigated. Further to the morphologic analysis of the macrophages on the PHMB-coated surfaces, the secretion of cytokines and the formation of reactive oxygen species were analyzed.

Additionally, to mimic pre-operative implant contaminations, a co-culture system was set up. For that, THP-1 derived macrophages were cultured on PHMB-coated surfaces that were contaminated with *S. epidermidis* and *P. aeruginosa* - microorganisms that predominantly colonize implant devices - before. Cell morphology and cytokine secretion were analyzed afterwards.

## Materials and methods

### Test specimen and surface treatment

Test specimens of Ti6Al4V alloy material with a diameter of 1.1 cm and a thickness of 2 mm (DOT GmbH medical implant solutions, Rostock, Germany) were used. The surface was either oxidized by incubation of each disc in 1.0 ml 5% H_2_O_2_ for 24 h (H-surface) [26], protected from light, or by alkaline treatment in 2.0 ml pre-heated 5 M NaOH (60 °C) for 2 h (N-surface) [27]. Treatment was conducted in 24-well cell culture plates. After washing in 1.5 ml sterile water (B.Braun Melsungen AG, Melsungen, Germany) four times for 5 min each, specimens were incubated in 1.0 ml PHMB (Fagron GmbH&Co. KG, Barsbüttel, Gremany) solution (30 μg/ml) in PHMB-pre-coated 24-well plates. NaOH-activated specimens were incubated for 0.5 h, 1 h, and 2 h (NP-surfaces), and H_2_O_2_-treated test specimens for 24 h (HP-surface). To estimate the amount of adsorbed PHMB after incubation of the test specimens, 250 μl of the coating solution with residual PHMB was spectrophotometrically determined at 235 nm. With the help of a calibration curve, the amount of adsorbed PHMB was calculated. To set up the calibration curve, 250 μl of PHMB solution with increasing concentrations (0–30 μg/ml) were added to a 96 well quartz plate and extinction at 235 nm was measured (Fig. S1).

PHMB-coated surfaces after NaOH-treatment contained approximately 5 μg, 7 μg and 10 μg PHMB/cm^2^ (NP5, NP7, NP10), whereas H_2_O_2_-oxidized specimen adsorbed 4 μg PHMB/cm^2^ (HP4-surface). The thickness of the PHMB film is below 10 nm.

### Cell culture and differentiation

Human leukemic THP-1 monocytes (Sigma Aldrich, Darmstadt, Germany) were grown in RPMI1640 (PAN-Biotch GmbH, Aidenbach, Germany), supplemented with 10% (v/v) fetal bovine serum (FBS) and 2 mM L-glutamine in a humidified atmosphere (37 °C, 5% CO_2_). The cells were subcultured twice a week and the morphology was checked regularly. The cell lines were free of mycoplasmas. The cell viability was assessed by trypan blue exclusion.

The THP-1 monocytic cells were differentiated with phorbol 12-myristate 13-acetate (PMA; Sigma Aldrich, Darmstadt, Germany) to unpolarized M0 macrophages in cell culture flasks. Concentrations of 25, 50 and 100 ng/ml PMA as well as different incubation periods (24 h, 48 h, 72 h) were tested. Afterward, cells were washed twice with phosphate buffered saline without Ca^2+^/Mg^2+^ (w/oPBS), trypsinized and used directly for further experiments without further addition of PMA.

### Immunofluorescent labeling and confocal microscopy

The cell surface molecules CD14 and CD68 were stained for fluorescent microscopy. After differentiation with PMA (25 ng/ml) for 48 h, cells were trypsinized, seeded onto test specimens and cultured for 2 h in cell culture medium without PMA. Afterwards, the specimens with adhered cells were washed with wPBS and incubated in 1.5% paraformaldehyde for 10 min followed by two washing steps with 1% (w/v) BSA in wPBS. Subsequently, the cells were incubated in wPBS/BSA with 1% sodium azide for 10 min. After washing, cells were incubated with 50 μl (10 μg/ml) primary mouse anti-human antibodies CD14 (301,801, BioLegend, San Diego, CA, USA) and CD68 (333,801, BioLegend, San Diego, CA, USA) for 1 h at 4 °C. Primary antibodies were labeled with a secondary AlexaFluor® 488 goat anti-mouse IgG antibody (50 μl, 2 μg/ml, 405,319, BioLegend, San Diego, CA, USA) while protected from light for 1 h. Before fluorescent microscopy with a confocal laser scanning microscope (Zeiss Axio Observer Z.1), cells were washed twice in wPBS.

### Cell adherence

Undifferentiated monocyte cells were adjusted to 2 × 10^6^ cells/ml and PMA (25 ng/ml) was added. A volume of 50 μl was applied to each of the test specimens following 0.5 h, 1 h and 2 h incubation in a humidified atmosphere. Additionally, cells without PMA treatment were cultured on the test specimens.

Afterwards, the specimens were washed in 1.0 ml fresh cell culture medium in a 24-well cell culture plate and immediately transferred again into a new well with 1.0 ml medium.

Non-adherent cells in the supernatant were detected by determination of ATP (BacTiter-Glo, Promega, Madison, WI, USA). For that, 100 μl of the supernatant were transferred into a white 96-well plate and immediately 100 μl of BacTiter-Glo™ reagent were added. After incubation for 10 min, the luminescence signal was measured (Multimode reader LB941 TriStar, Berthold Technologies, Bad Wildbad, Germany). Using a calibration curve, possibly loosened cells in the supernatants were quantified.

### Intracellular reactive oxygen species

THP-1 monocytes were differentiated to macrophages (25 ng/ml PMA, 48 h) in T75 cell culture flasks and harvested by trypsinization. After subsequent centrifugation, the pellet was suspended in cell culture medium. Cells were adjusted to 2 × 10^6^ cells/ml, and 50 μl of the suspension were applied to each of the test specimens. In addition to untreated control test specimens, NaOH- and H_2_O_2_-treated surfaces as well as PHMB-coated surfaces were used. A 10 mM Menadione solution (2-methyl-1,4-naphthoquinone, Sigma Aldirch, Darmstadt, Germany) served as positive control.

The variously processed Ti6Al4V disks with adherent cells were placed on wet cellulose flakes (Rotilabo® test flakes) in a 24-well cell culture plate. After culture for 2 h in a humidified atmosphere, the supernatant was removed and 50 μl CellRox® green reagent (Invitrogen, Carlsbad, CA, USA) working solution were added following incubation for 30 min. To obtain the working solution, CellROX® reagent was diluted as given by the manufacturer in cell culture medium. Afterwards, cells were washed twice with wPBS and lysed with 300 μl NaOH (0.1 M) for 10 min. Thereafter, 200 μl of the cell lysate were transferred into a black 96-well microtiter plate. Fluorescence was measured with an excitation wavelength of 490 nm and emission wavelength of 535 nm (Multimode reader LB941 TriStar, Berthold Technologies, Bad Wildbad, Germany).

### Macrophage cell viability on test specimens

Macrophages, pre-incubated with PMA (25 ng/ml) for 48 h were seeded on test specimens (100 μl, 1 × 10^6^ cells/ml) placed on wetted filter discs following cell culture for 24 h. Next to control test specimens, specimens coated with PHMB after alkaline treatment (5, 7 and 10 μg PHMB/specimen) and H_2_O_2_-treatment (4 μg PHMB/specimen) were used. As positive control, LPS (100 ng/ml) was applied. After 24 h, supernatants were transferred to microreaction tubes, centrifuged for 3 min at 4 °C (34,000 x g) and stored at − 20 °C until use for cytokine measurement. Fresh medium was added to the cells and incubation was continued for 24 h, following again a medium change and incubation for 24 h.

Furthermore, macrophages on control specimens were cultured under addition of PHMB (1, 2 and 4 μg) in solution for 24 h.

Cell viability of adhered cells was measured after 24 h, 48 h and 72 h using 2,3-Bis-(2-methoxy-4-nitro-5-sulfophenyl)-2H-tetrazolium-5-carboxanilid (XTT disodium salt, AppliChem, Darmstadt, Germany). The test specimens were transferred into 1.0 ml of fresh cell culture medium and 500 μl of XTT solution (1 mg/ml in cell culture medium, 25 μM phenazine methosulfate) were added. After 3 h of incubation, absorbance was measured at 450 nm and cell viability was calculated as % of control. After cell viability analysis, actin filament staining was performed.

### Co-culture medium, bacterial contamination and subsequent cell culture

The Gram-positive slime-producing microorganism *S. epidermidis* ATCC 35984 and the Gram-negative *P. aeruginosa* ATCC 27853 were used as test microorganisms. A bacterial subculture was plated on Trypticase soy agar (TSA) plates following incubation at 37 °C for 24 h. The bacteria were harvested by rinsing the agar plate with 2.0 ml saline solution following centrifugation at 13,000 rpm for 1 min. Cells were washed once with 1.0 ml 1% Tween 80 to disrupt cell clusters and twice with wPBS. After final centrifugation at 5,000 rpm for 3 min, bacteria were resuspended in co-culture medium. The optical cell density (OD) of the suspension was adjusted to 0.30–0.35 (*S. epidermidis*) and 0.11–0.15 (*P. aeruginosa*) at 620 nm, respectively, to reach a bacterial density of 1-3 × 10^8^ cfu/ml.

For the co-culture experiments, a special co-culture medium was applied consisting of cell culture medium with addition of 10% tryptic soy broth [27, 28]. The initial bacterial suspension was diluted in this medium to 1-3 × 10^5^ cfu/ml.

A volume of 50 μl of the bacterial suspension was applied to the test specimens (control, NP with 5 μg, 7 μg or 10 μg PHMB) that were placed on wetted cellulose flakes. After incubation for 6 h at 37 °C, the supernatant was removed and 100 μl of a macrophage cell suspension (1 × 10^6^ cells/ml) were added following cell culture for 24 h in a humidified atmosphere. Next, the supernatants were removed, centrifuged at 13,000 rpm and stored at − 20 °C until cytokine measurement. Fresh medium (100 μl) was added to the cells and incubation was continued for further 24 h. The medium was renewed and cells were incubated again for 24 h. The supernatants again were stored for cytokine measurement. Simultaneously, cell viability analysis and actin filament staining were performed at day 1, 2 and 3 of cell culture.

For determination of reduction factor (rf), bacteria incubated on further test specimens for 6 h on PHMB-uncoated and -coated surfaces were removed from the surface. For that, the specimens were placed in a 24-well cell culture plate with 1.0 ml NaCl solution and glass beads and incubated while shaking for 2 min. Afterwards, the suspension was plated on trypticase soy agar plates. After counting, rf were calculated by following formula: rf = lg_nc_ – lg_nd_, where rf is the lg bactericidal reduction factor, nc is the number of cfu/ml from control plates, and nd is the number of cfu/ml after exposure to the PHMB coated surface. The rf is calculated as mean factor from three to five independent experiments.

### Actin filament staining

Specimens with adherent cells were washed and cells fixed by addition of 1.5% paraformaldehyde. Afterwards, cells were washed with wPBS twice and incubated in 0.1% Triton X-100 for 15 min. After washing, Molecular Probes™ Image-iT™ FX signal enhancer (Invitrogen, Carlsbad, CA, USA) was added and let stand for 30 min. The solution was removed and Alexa Fluor® 488-Phalloidin (Invitrogen, Carlsbad, CA, USA) in wPBS with1% BSA was applied. After 60 min incubation, the test specimens were washed with wPBS twice and dried. For confocal laser scanning microscopy, an argon laser (488 nm) with a band-pass filter (505–530 nm) was used.

### Cytokine detection

First, the secretion of TNFα and IL-6 was analyzed after culture of THP-1 macrophages on Ti6Al4V specimens, contaminated with *S. epidermidis* and *P. aeruginosa* before, using ELISA assay (PeproTech, Hamburg, Germany). The assay was conducted as given by the manufacturer. After each step, a washing step was included. In short, capture antibody was incubated in 96-well plates at 4 °C overnight; block buffer was added and incubated for 1 h at room temperature. Then, the samples and standards were incubated for 2 h in the micro well plate. Following the next washing step, an Avidin-HRP conjugate was added and incubation was performed for 30 min. Color development after reaction with ABTS substrate was analyzed after 25 min.

To study further secreted cytokines associated with inflammation of THP-1 macrophages after cultivation following a contamination with *S. epidermidis*, the LEGENDplex™ human M1/M2 macrophage and the human inflammation panel (BioLegend, San Diego, CA, USA) were used and conducted as given by the manufacturer. In short, the stored supernatants were incubated overnight with assay buffer and capture beads while shaking at 4 °C. The samples were washed and detection antibody was added. After subsequent incubation for 1 h while shaking at room temperature, streptavidin-phycoerythrin was applied and samples were incubated again for 30 min while shaking. After washing, 100 μl of wash buffer were added to the samples. Measurement and analysis were done with BD FACSCanto, FACSDiva 8.0.2 and LEGENDplex™ software.

### Statistical analysis

Quantity changes of reactive oxygen species between control surfaces and treated surfaces (fold change) were represented as mean + SD. The experimental reproducibility was assured by conducting three to six independent trials consisting of two technical replicates each. The statistical significance was tested with the Kruskal-Wallis test following Dunn’s comparison.

For the LEGENDplex™ assay, minimum five independent experiments were performed, each comprising two technical replicates. Statistical analysis was done with Kruskal-Wallis tests following Dunn’s comparison and two-way ANOVA followed by Bonferroni post test, respectively.

## Results

### Monocyte differentiation and cell adherence

**Fig. 1:**
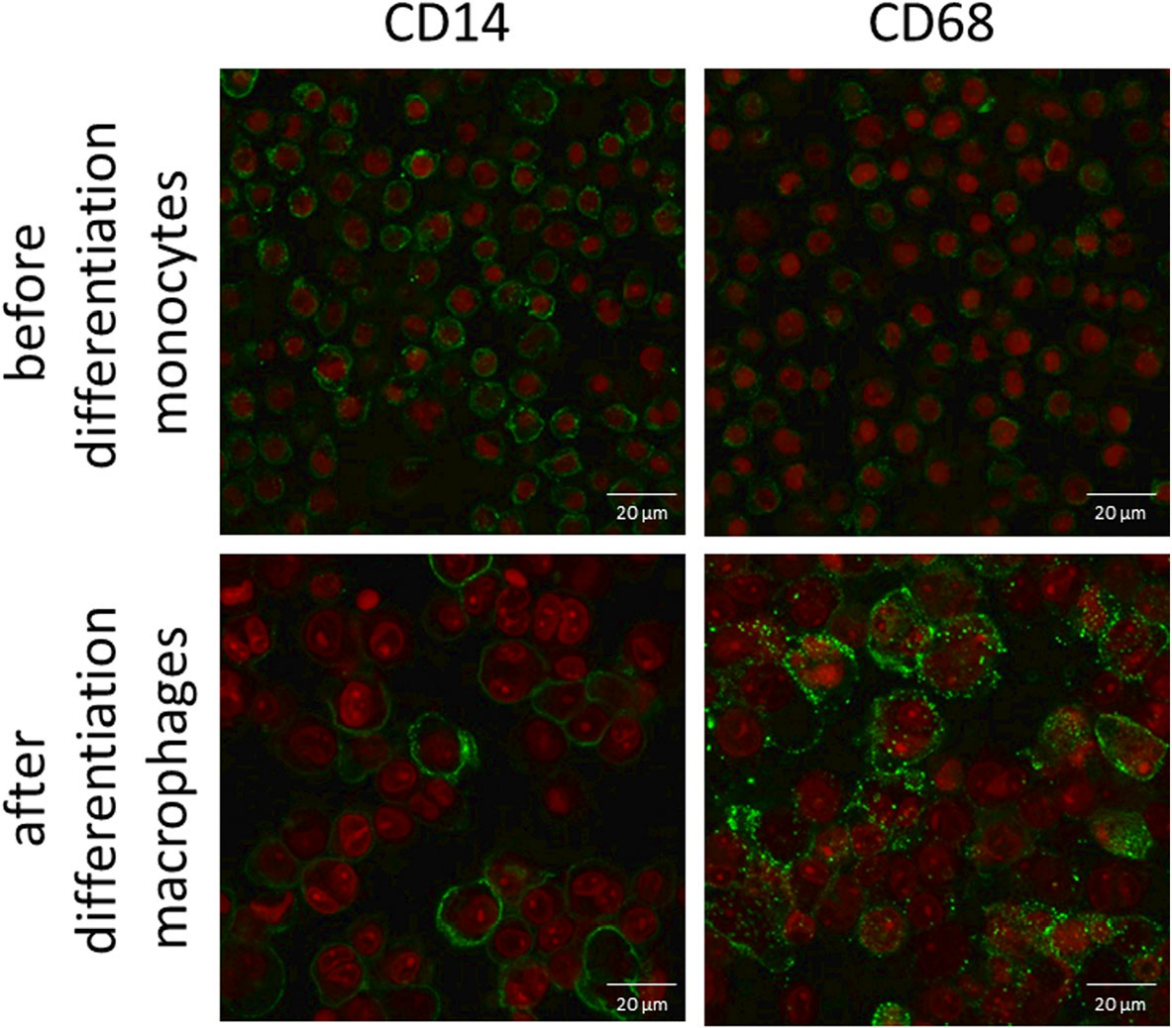
Monocyte differentiation of THP-1 cells to M0 macrophages. Representative confocal microscopic images of cells immune labeled (green) for CD14 (left panels) and CD68 (right panels) and stained cell nuclei with PI (red), before (upper panels) and after (lower panels) treatment with PMA; 40x magnification

An incubation period of 48 h while adding 25 ng/ml PMA was selected as an adequate parameter for differentiation of THP-1 monocytic cells to unpolarized M0 macrophages. The macrophages became adherent and cell spreading increased (Fig. 1). The expression of the macrophage marker CD68 on the differentiated cells was rising compared to undifferentiated monocytes (Fig. 1). Additionally, the CD14 marker was more clearly expressed on the monocytic cells than on the differentiated macrophages (Fig. 1), which confirmed successful differentiation.

Independently of the tested incubation times (30 min, 1 h and 2 h), 100% of the cells treated with PMA adhered to the surface of control specimens, on H- and N-processed and on PHMB-coated HP- and NP-surfaces. Control cells without PMA treatment did not adhere to all tested surfaces.

### Analysis of cell differentiation and morphology on PHMB-coated surfaces

**Fig. 2:**
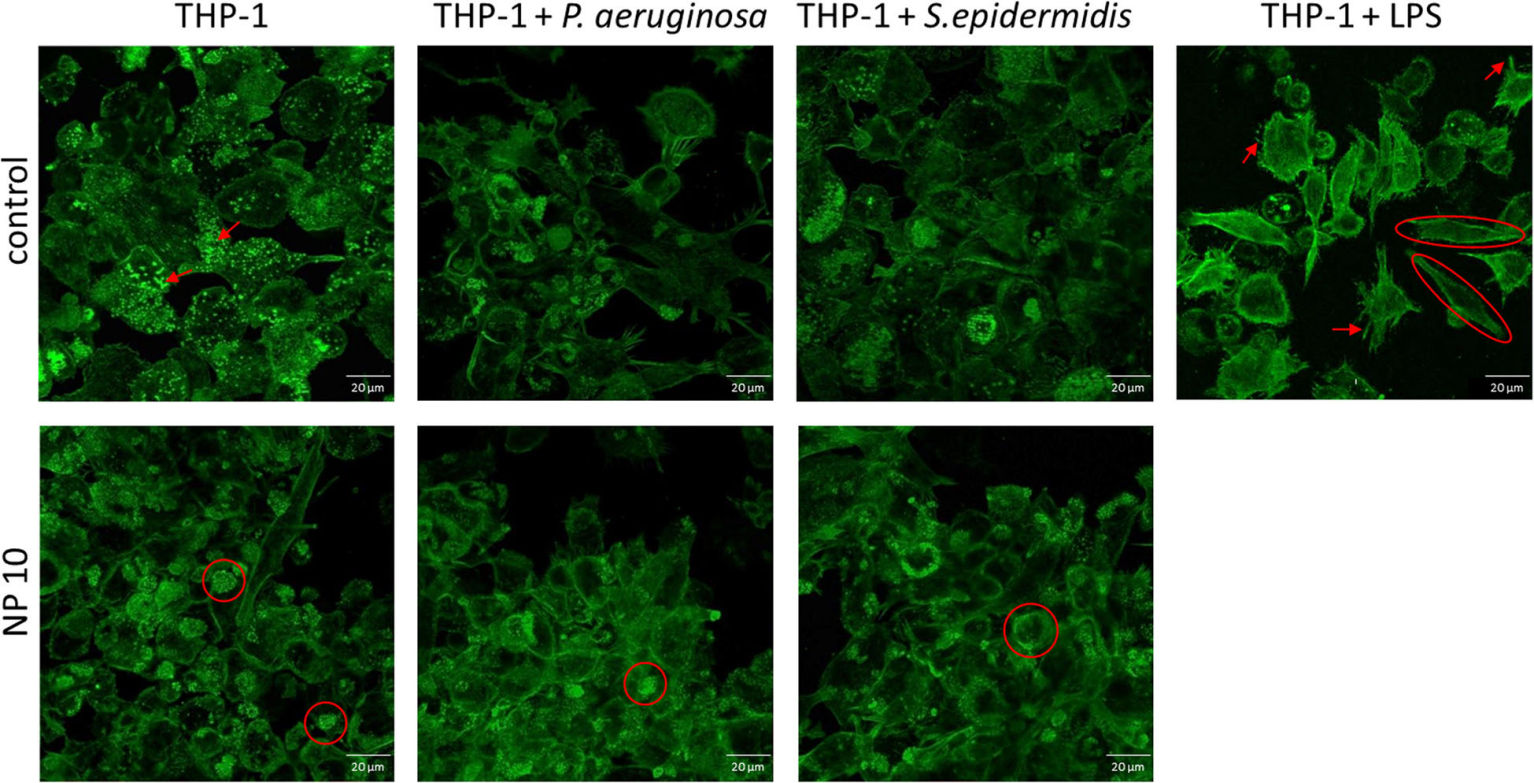
Cell morphology of macrophages differentiated before attachment to test specimen with and without PHMB-coating (NP10). Cultivation for 24 h with and without contamination of test specimens with *S.epidermidis* and *P.aeruginosa* prior to cell attachment

Macrophages differentiated before contact to the test specimen show a spreaded shape for up to 72 h on untreated Ti6Al4V surfaces (Fig. 2). Podosomes and stress fibers were visible over the whole incubation period. LPS treatment led to more ellipsoid cell spreading and development of filopodia. PHMB-coating resulted in a more rounded shape of the cells with less podosomes and stress fibers. Contamination of uncoated Ti6Al4V-surfaces with *S. epidermidis* or *P. aeruginosa* led to slightly stronger cell spreading but less podosome formation. The contamination of surfaces with antimicrobial coating and previous contamination did not result in changes in actin filaments in comparison to non-contaminated coated surfaces (Fig. 2).

### Intracellular reactive oxygen species

**Fig. 3:**
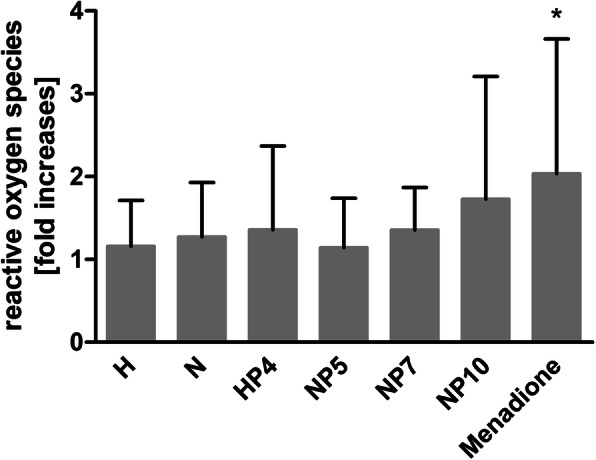
Fold increases of reactive oxygen species (iROS) generated after 2 h incubation of macrophages on variously treated Ti6Al4V implant surfaces (H = H_2_O_2_-oxidized, N = NaOH-treated, HP/NP = PHMB coated after H_2_O_2_- or NaOH-processing with adsorbed amounts of PHMB) analyzed with CellROX® green reagent. Signal intensity (mean + SD for *n* = 3–6) is represented as fold increase of iROS in cells on untreated Ti6Al4V surfaces

Cultivation of macrophages on H- and N-surfaces induced only slight, non-significant increases in iROS (Fig. 3). The PHMB-coated H_2_O_2_-oxidized surface (HP) containing 4 μg PHMB increased reactive oxygen species generation 1.36 times. Similar amounts of PHMB on the NaOH-treated surface (NP) increased the iROS generation only 1.14 times. The coating of NaOH-processed surfaces with 7 and 10 μg PHMB produced a 1.36- and 1.73-fold increase of iROS, respectively. The positive control Menadione doubled the amount of iROS.

As observed, the CellRox® treatment of cells incubated previously with the positive control Menadione, led to loosening and detachment of cells, resulting in a low signal.

### Antimicrobial efficacy

**Fig. 4:**
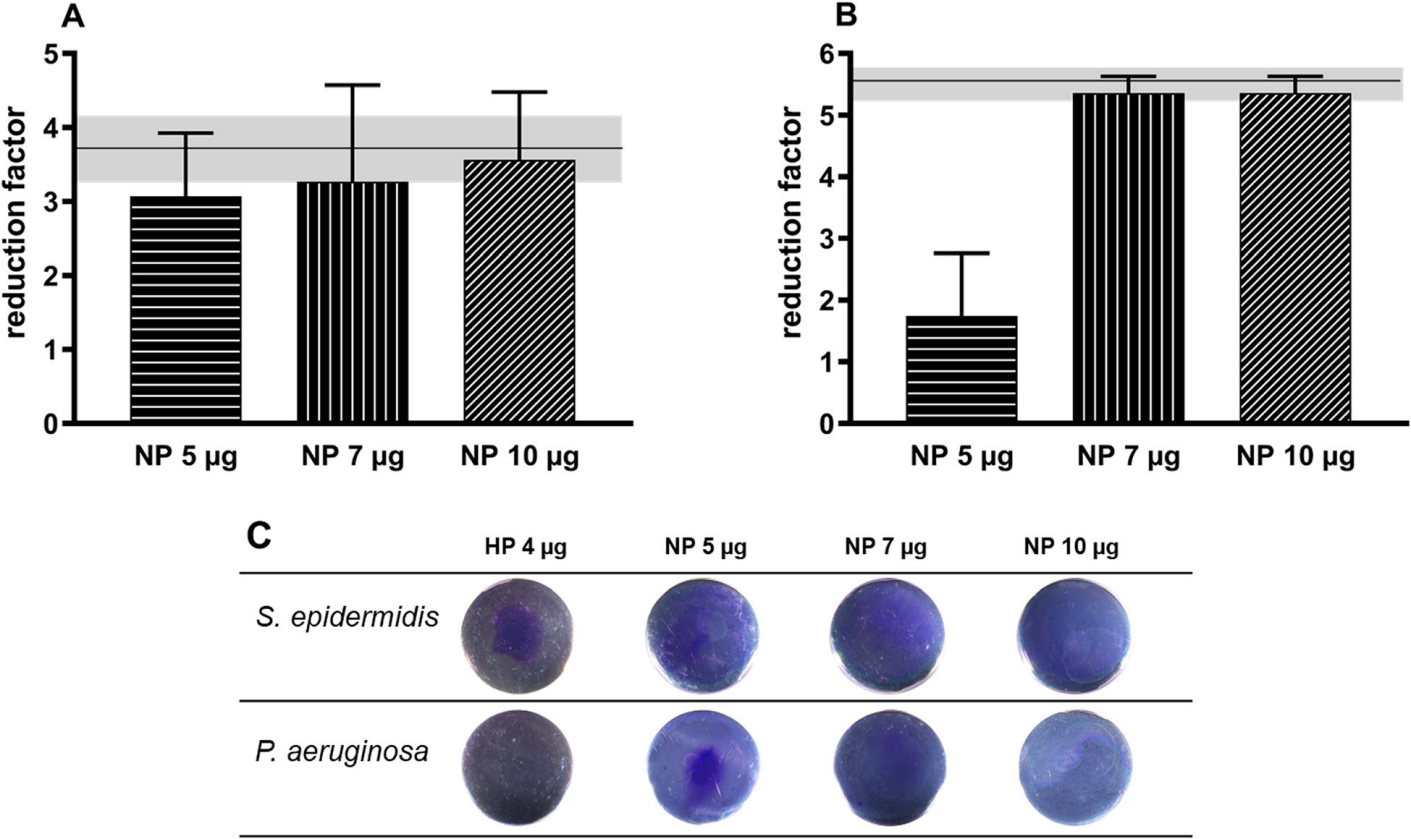
Log10 reduction factors of **A**) *S. epidermidis* and **B**) *P. aeruginosa* incubated for 6 h on PHMB-coated Ti6Al4V specimens with different PHMB concentrations. The horizontal line illustrated the mean of counts (±SD) of living microorganisms on control specimens. **C** Crystal violet staining of bacteria after incubation on PHMB-coated test specimens. An adherence of bacteria was observed on the HP4 (*S. epidermidis*) and the NP5 (*P. aeruginosa*) surfaces

PHMB-coated test specimen (NP5, NP7, NP10) were treated with 50 μl of a 1 × 10^5^ cfu/ml bacterial suspension of *S. epidermidis* and *P. aeruginosa*, respectively, for 6 h. After the incubation on the NP10 surface, nearly no viable microorganisms could be detected in the plated volume, reflected by reduction factors (rf) of 3.57 and 5.36 (Fig. 4). The NP7 surface also led to high reduction factors (3.27 and 5.36 respectively). The coating with 5 μg PHMB led to a high reduction of *S. epidermidis* (rf = 3.07) but only to a slight reduction of *P. aeruginosa* (rf = 1.75). Reduction factors for the reduction on the HP surface with 4 μg PHMB were already published by Hornschuh et al. (2019) [24]. The reduction factor for *S. epidermidis* on the HP4 surface was about 3.84 ± 0.34 log levels. *P. aeruginosa* was reduced by 5.6 ± 0.26 log levels.

### Viability of macrophages on different surfaces

**Fig. 5:**
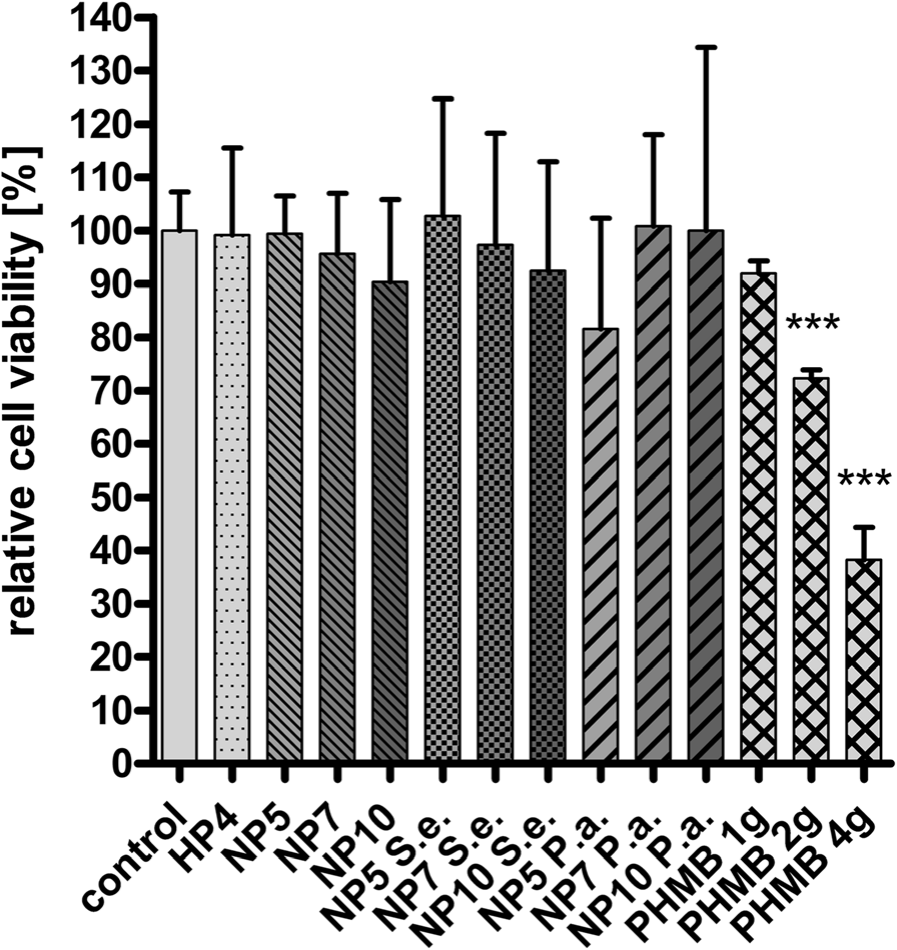
Viability of macrophages [%] after 24 h culture on variously treated Ti6Al4V surfaces (H = H_2_O_2_-oxidized, N = NaOH-treated, HP/NP = PHMB coated after H_2_O_2_- or NaOH-processing) with adsorbed amounts of PHMB [μg], and controls with free PHMB as well as contamination with *S.epidermidis*. (S. e.) and *P. aeruginosa* (P. a.). Cell viability (mean + SEM for *n* = 3–6) was measured using XTT-assay. ***p < 0.001

Adsorbed PHMB on the specimen surface did not cause changes in the relative viability of macrophages cultured for 24 h (Fig. 5), 48 h or 72 h, but 2 μg and 4 μg PHMB in solution decreased the cell viability significantly already after 24 h (Fig. 5). The contamination of PHMB-coated test specimen with *S. epidermidis* or *P. aeruginosa* led to no changes in cell viability in comparison to coated and uncoated surfaces. The viability of macrophages on uncoated, contaminated surfaces was not measurable, but a biofilm formation on the test specimen was observed.

### Cytokine secretion

**Fig. 6:**
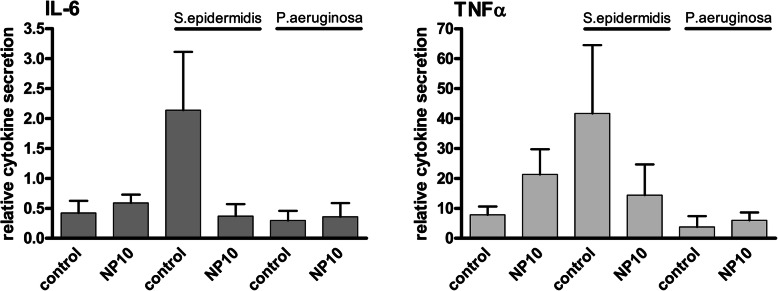
Cytokine secretion of THP-1 macrophages cultured on PHMB-coated and uncoated Ti6Al4V specimens with and without contamination with *S.epidermidis* and *P.aeruginosa*. No significant increase in cytokines was detectable, but *S. epidermidis* rised cytokine secretion of both TNFα and IL-6

First, the secretion of cytokines after contamination of the test specimens with *S. epidermidis* and *P. aeruginosa* was analyzed using ELISA assay (Fig. 6). The coating with PHMB (10 μg) had slight increasing effects on TNFα secretion and no effects on IL-6 secretion. A contamination with *S. epidermidis* led to strong increases in both IL-6 and TNFα-secretion. The coating with PHMB reduced this secretion to a normal level. The contamination with *P. aeruginosa* led not to increases in cytokine secretion.

**Fig. 7:**
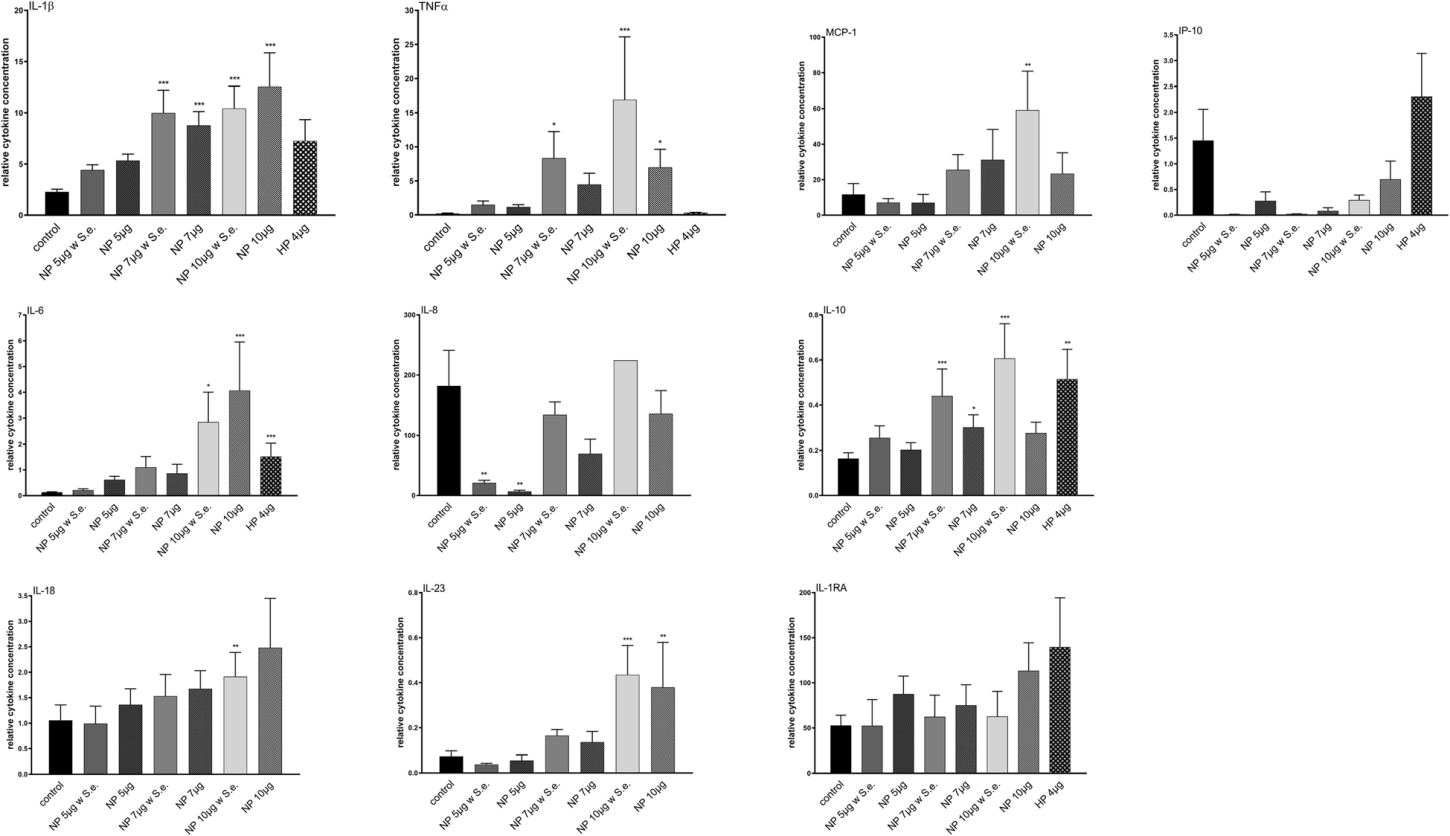
Relative concentrations of secreted cytokines (mean + SEM for *n* = 4–6) normalized to cell viability after 24 h incubation of macrophages on variously treated Ti6Al4V surfaces (H = H_2_O_2_-oxidized, N = NaOH-treated, HP/NP = PHMB coated after H_2_O_2_- or NaOH-processing with adsorbed amounts of PHMB in brackets, and controls with free PHMB). *p < 0.05; **p < 0.01; ***p < 0.001; ****p < 0.0001 significantly different from the control

For further experiments, only a contamination with *S. epidermidis* was conducted. For macrophages cultured on the HP surface, the secretion of six cytokines (TNFα, IL-6, IL-10, IL-1β, IL-1RA, IP-10) was analyzed after 24 h (Fig. 7, Table 1). Cytokine secretion after cultivation on different NP surfaces (5 μg, 7 μg, 10 μg) without and with contamination with *S. epidermidis* (6 h) was analyzed after 24 h, 48 h and 72 h for ten cytokines (TNFα, IL-6, IL-10, IL-1β, IL-1RA, IP-10, CCL-2, IL-8, IL-18, IL-23).

**Table 1:**
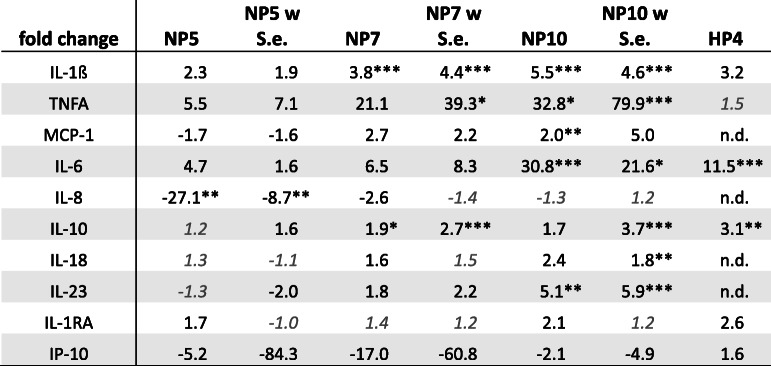
Fold changes of relative cytokine concentrations after 24 h cultivation of THP-1 derived macrophages on PHMB-coated Ti6Al4V surfaces. *p < 0.05; **p < 0.01; ***p < 0.001; values <|-1.5| are considered as no changes and displayed in italics and grey, n.d. not determined. w S.e. = with previous *S. epidermidis* contamination, NP/HP PHMB-coated surface after NaOH or H_2_O_2_-treatment

The cytokine secretion was highest after 24 h of incubation following a rapid decrease (data not shown). Neither the oxidized H-surface nor the alkaline-processed N-surface induced significant changes in cytokine secretion in comparison to that of the control surface (data not shown).

In comparison to the control surface, the HP-surface led to increases in the secretion of all measured cytokines after 24 h: IL-1β (3.2-fold increase), TNFα (1.5-fold), IL-6 (11.5-fold), IL-10 (3.1-fold), IL-1RA (2.6-fold) and IP-10 (1.6-fold).

On NP-surfaces, the more PHMB was adsorbed, the higher was the cytokine secretion (Table 1).

The NP-surface containing 5 μg PHMB was the one with the least effect on cytokine secretion. A previous contamination with *S. epidermidis* hat just slight effects on cytokine secretion in comparison to non-contaminated, but PHMB-coated surfaces.

## Discussion

As joint replacement leads to the attachment of macrophages on the implant surface, it is recognized as a foreign body by these cells and an acute inflammatory process is initiated by the secretion of inflammatory cytokines [36–38]. Simultaneously, the differentiation of mesenchymal stem cells to osteoblasts is inhibited [37, 39–41]. If this acute inflammation cannot be dissolved, chronic inflammation can occur, leading to aseptic implant loosening. This reaction is initiated by M1 macrophages, that secrete pro-inflammatory cytokines (e.g. IL-6, TNFα) and ROS, leading to integration failure via tissue damage [42–45]. Additionally, the secreted pro-inflammatory cytokines contribute to the activation of NFκB and recruit inflammatory cells to the site of injury, promoting the polarization of T cells to Th1-cells [42, 43, 46]. In contrast, M2 macrophages function as inhibitors of the initial inflammatory process [43, 47, 48], leading to increased osseointegration [49–51] by suppressing the production of pro-inflammatory cytokines and secreting anti-inflammatory cytokines as IL-10 or IL-1 receptor type A [52–54].

THP-1 cells display a monocytic-like cell line with distinct monocytic markers [55]. Treatment with phorbol esters leads to differentiation to M0 macrophages, behaving similarly to native macrophages and thus providing a suitable model for studies of macrophage responses [56]. Their differentiation leads to secretion of various cytokines as IL-6, TNFα, IL-1β and IL-10 [57]. For these reasons, THP-1 monocytic-like cells were chosen as the model in the present study.

The successful differentiation of THP-1 using PMA was confirmed by fluorescence staining of CD14 and CD68, revealing the differentiation to macrophages. The morphology of differentiated cells was macrophage-like after treatment with PMA on PHMB-coated (HP, NP) and uncoated (H, N) surfaces.

A ﻿study of Kamendulis et al. revealed that the antimicrobial agent PHMB itself does not evoke immune cell activation. An incubation of RAW264.7 mouse macrophages under stimulation with PHMB neither led to activation of the cells nor to increased proliferation [58]. In our study, we neither regarded a cell activation of monocytes cultured on PHMB-coated surfaces, confirming the findings of Kamendulis et al.

The contamination with microorganisms and a following biofilm formation impedes cell attachment and thus an osseointegration of the implant. The coating with PHMB prevents the biofilm formation by contact killing of bacteria immediately after their attachment to the implant surface. Thus, a possible contamination that can occur during the surgery from the depth of the skin is prevented, enabling cell attachment and osseointegration. In our study, on uncoated and contaminated specimens a biofilm formation was visible, that impeded macrophage attachment. In contrast, PHMB-coated specimens enabled macrophage attachment and viability by previous killing of microorganisms.

However, microenvironmental stimuli as a surface coating can increase the generation of iROS. Reactive oxygen species cause an increased passage of inflammatory cells from the blood to the tissue what promotes tissue injury [59]. iROS that overload the cell’s antioxidant response can result in further inflammation and cytotoxicity. As shown by Kim et al., PHMB induces just slight increases of 1.39-fold in iROS after treatment of A549 cells with PHMB (20 μg/ml) for 3 h, which is assumed to be a non-toxic level of ROS [60]. Our study also demonstrated only slight increases in iROS after cultivation of macrophages on PHMB-coated HP- and NP-surfaces for 2 h. Only amounts of > 7 μg PHMB/specimen on alkaline treated implant material led to slightly higher amounts of iROS. In contrast, the kind of pretreatment of the surface before PHMB adsorption did not have an effect on iROS generation. The positive control Menadione showed only minor effects, due to detachment of cells after CellROX® treatment. Regarding these results, the PHMB coating may not evoke increasing inflammatory effects.

However, a main reason for inflammatory processes are cytokines, secreted by macrophages. Based on the cytokine profile secreted by macrophages, it is possible to discriminate between M1 and M2 macrophages. M1 macrophages secrete high levels of inflammatory cytokines as IL-6, IL-1β and TNFα, whereas M2 macrophages express anti-inflammatory cytokines as IL-10 or IL-1RA [61]. By M1 macrophages secreted pro-inflammatory cytokines contribute to the activation of NFκB and cause an influx of inflammatory cells to the site of injury [42, 43, 46]. These reactions result in inflammation and bone resorption [62], promoting implant failure.

After a treatment of human colon adenocarcinoma cells (Caco-2), mouse neural cells (N2-A) and human hepatocellular carcinoma cells (HepG2) with PHMB (80 μg/ml) for 12 h, Creppy et al. revealed mainly slight, non-significant increases in cytokine levels [34]. But, as for iROS generation, also microenvironmental stimuli as the physical surface properties of an implant can also have an influence on the differentiation and polarization of monocytes to pro-inflammatory M1 and anti-inflammatory M2 macrophages [50, 51, 63–66], explaining why the surface of an implant is critical for successful osseointegration [67, 68]. In a study of Hotchkiss et al. a reduction of pro-inflammatory cytokine secretion (IL-1β, IL-6, TNFα) of primary murine macrophages after the chemical and mechanical treatment of a TiZr-alloy is stated [51]. The treatment of a titanium surface with ozone also led to a reduction in the pro-inflammatory cytokines TNFα and IL-6 by LPS stimulated macrophages [69].

In our study, we proved the cytokine secretion of M0 macrophages incubated on uncoated and PHMB-coated surfaces without and with previous bacterial contamination. Even if due to the antimicrobial effect of the surface coating nearly no viable microorganisms remain on the implant surface, residual bacterial cell wall debris and other fragments might have an impact on cytokine secretion. Nevertheless, cytokine secretion was just slightly increased in contrast to non-contaminated PHMB-coated surfaces. Our study revealed the smallest impact on cytokine levels in comparison to uncoated Ti6Al4V surfaces for the NP5 surface. Interesting is the strong reduction of IL-8 and IP-10 due to the NP5 and the NP7 surface. The IL-8 decrease indicates a downregulation of osteoclastogenesis [70] whereas the strong decrease in IP-10 secretion is a marker for a reduced interferon activity, thus indicating reduced activation of macrophages and antigen presentation.

In comparison to the NP surfaces, the HP surface led to a slightly different cytokine secretion pattern. The HP4 surface led to increased secretion of IL-10, IL-1RA, IL-6 and IP-10 giving reasons to believe in a shift of M0 macrophages to M2 macrophages. The high amounts of IL-10 as well as the reduced amounts of IL-8 additionally indicate a downregulation of osteoclastogenesis [70, 71]. Moreover, IL-10 secretion acts anti-inflammatory by inhibiting macrophages by negative feedback-loop, thus also decreasing TNFα production.

In comparison to the control surface, especially NP-specimen containing 5 μg PHMB and HP4-surfaces led to the lowest effects on the cytokine secretion pattern and minor inflammatory effects, respectively, and are thus considered as surfaces with a high cytocompatibility. The NP7 surface led to higher cytokine secretion in comparison to the NP5 surface. However, it is also considered as a possibly compatible coating, because of its higher antimicrobial efficacy in comparison to the NP5 surface. Additionally, the advantage of the NaOH treatment prior to PHMB coating lies in the adjustability of the adsorbed PHMB amount and the possibility to control the antimicrobial efficacy as well as the cytokine secretion. But, with nearly equal amounts of PHMB, the HP4 surface has an even higher antimicrobial activity in comparison to the NP5 surface with less changes in the cytokine secretion pattern of the macrophages.

However, also increasing pro-inflammatory cytokine levels were detected after incubation of macrophages on the HP4 surface. As shown by Lencel et al., who detected increases in alkaline phosphatase activity and calcium apatite formation due to rising TNFα concentrations, these effects might have a positive effects on osseointegration [72]. Additionally, an acute inflammatory process is also necessary for optimal wound healing, but a shift to chronic inflammation should be prevented. In what extend the shifts in cytokine secretion shown in our study have an overall pro- or anti-inflammatory effect has to be further evaluated.

## Conclusion

The kind of treatment of the implant surface for PHMB adsorption as well as the amount of adsorbed PHMB have an impact on macrophage reactions. The NP5-surface and the HP4-surface generally had a minor influence on the macrophages and should therefore be considered as the ones with the best cytocompatibility with macrophages tested in the present study. Cell adherence and cell morphology of macrophages were not impaired by PHMB. Additionally, a contamination of HP- and NP-surfaces with *S. epidermidis* did not show a significant effect on macrophage viability due to a high reduction in viable microorganisms by the PHMB-coating.

Whether the presented PHMB-coatings have a long-term impact on inflammatory reactions should be further evaluated to determine, if a chronic inflammatory reaction can be prevented by PHMB-coating. Additionally, to evaluate impacts on osseointegration, co-cultures with osteoblast-like cells should be performed to analyze alkaline phosphatase activity and calcium apatite formation. This would enable a more explicit recommendation for the use of PHMB as an antimicrobial coating on implant devices.

## Supplementary Information

**Additional file 1: Supplementary Fig. 1.** Calibration curve of PHMB in water. Two hundred fifty microlitre of aqueous PHMB solution with rising concentration were added to each well of a 96 well quartz plate and adsorption was measured at 235 nm. *N* = 4.

## Data Availability

The datasets during and/or analysed during the current study available from the corresponding author on reasonable request.

## References

[1] Fink B, Anagnostakos K, Winkler H (2019). Periprosthetic joint infection. Biomed Res Int.

[2] Tande AJ, Patel R (2014). Prosthetic joint infection. Clin Microbiol Rev.

[3] Fey PD, Olson ME (2010). Current concepts in biofilm formation of Staphylococcus epidermidis. Future Microbiol.

[4] Moran E, Masters S, Berendt AR, McLardy-Smith P, Byren I, Atkins BL (2007). Guiding empirical antibiotic therapy in orthopaedics: the microbiology of prosthetic joint infection managed by debridement, irrigation and prosthesis retention. J Infect.

[5] Grimberg A, Jansson V, Liebs T, Melsheimer O, Steinbrück A (2016). Endoprothesenregister Deutschland (EPRD) Jahresbericht 2015.

[6] Grimberg A, Jansson V, Liebs T, Melsheimer O, Steinbrück A (2017). Endoprothesenregister Deutschland (EPRD) Jahresbericht 2016.

[7] Busscher HJ, van der Mei HC, Subbiahdoss G (2012). Biomaterial-Associated Infection: Locating the Finish Line in the Race for the Surface. Sci Transl Med.

[8] Kyomin C, Young JW, Kijun P, et al. Antibacterial infection and immune-evasive coating for orthopedic implants. Sci Adv. 2020;6:eabb0025. 10.1126/sciadv.abb0025 PMC7608784 33115733

[9] Tang P, Zhang W, Wang Y (2011). Effect of Superhydrophobic surface of titanium on *Staphylococcus aureus* adhesion. J Nanomater.

[10] Ferrari M, Cirisano F, Morán MC (2019). Mammalian Cell Behavior on Hydrophobic Substrates: Influence of Surface Properties.

[11] Chouirfa H, Bouloussa H, Migonney V, Falentin-Daudré C (2019). Review of titanium surface modification techniques and coatings for antibacterial applications. Acta Biomater.

[12] Yarramaneni V, Narayan A, Sachdeva A, Balakrishnan D, Prabhu N (2016). Emerging antibacterial coated dental implants, a preventive measure for Peri-implantitis. World J Dentist.

[13] Chainer J (2001). Home steel home - AK steel partners with AgIONTM to build world's first antimicrobial steel house. AISE Steel Technol.

[14] Kalyon BD, Olgun U (2001). Antibacterial efficacy of triclosan-incorporated polymers. Am J Infect Control.

[15] Raad I, Darouiche R, Hachem R, Sacilowski M, Bodey GP (1995). Antibiotics and prevention of microbial colonization of catheters. Antimicrob Agents Chemother.

[16] Hickok NJ, Shapiro IM (2012). Immobilized antibiotics to prevent orthopaedic implant infections. Adv Drug Deliv Rev.

[17] Cieplik F, Jakubovics NS, Buchalla W, Maisch T, Hellwig E, Al-Ahmad A (2019). Resistance toward chlorhexidine in Oral Bacteria - is there cause for concern?. Front Microbiol.

[18] Fritz SA, Hogan PG, Camins BC (2013). Mupirocin and chlorhexidine resistance in Staphylococcus aureus in patients with community-onset skin and soft tissue infections. Antimicrob Agents Chemother.

[19] Ledder RG, Gilbert P, Willis C, McBain AJ (2006). Effects of chronic triclosan exposure upon the antimicrobial susceptibility of 40 ex-situ environmental and human isolates. J Appl Microbiol.

[20] Silver S (2003). Bacterial silver resistance: molecular biology and uses and misuses of silver compounds. FEMS Microbiol Rev.

[21] Wuertz S, Miller CE, Pfister RM, Cooney JJ (1991). Tributyltin-resistant bacteria from estuarine and freshwater sediments. Appl Environ Microbiol.

[22] Kampf G (2018). Biocidal agents used for disinfection can enhance antibiotic resistance in gram-negative species. Antibiotics (Basel).

[23] Peeters E, Hooyberghs G, Robijns S (2018). An antibiofilm coating of 5-aryl-2-aminoimidazole covalently attached to a titanium surface. J Biomed Mater Res B Appl Biomater.

[24] Ferreira L, Zumbuehl A (2009). Non-leaching surfaces capable of killing microorganisms on contact. J Mater Chem.

[25] Kramer A, Eberlein T, Müller G, Dissemond J, Assadian O (2019). Re-evaluation of polihexanide use in wound antisepsis in order to clarify ambiguities of two animal studies. J Wound Care.

[26] Müller G, Benkhai H, Matthes R (2014). Poly (hexamethylene biguanide) adsorption on hydrogen peroxide treated Ti–Al–V alloys and effects on wettability, antimicrobial efficacy, and cytotoxicity. Biomaterials.

[27] Hornschuh M, Zwicker P, Schmidt T, Finke B, Kramer A, Muller G (2019). Poly (hexamethylene biguanide), adsorbed onto Ti-Al-V alloys, kills slime-producing staphylococci and Pseudomonas aeruginosa without inhibiting SaOs-2 cell differentiation. J Biomed Mater Res B Appl Biomater.

[28] Hornschuh M, Zwicker P, Schmidt T, Kramer A, Muller G (2020). In vitro evaluation of contact-active antibacterial efficacy of Ti-Al-V alloys coated with the antimicrobial agent PHMB. Acta Biomater.

[29] Ikeda T, Ledwith A, Bamford CH, Hann RA (1984). Interaction of a polymeric biguanide biocide with phospholipid membranes. Biochim Biophys Acta.

[30] Ikeda T, Tazuke S, Watanabe M (1983). Interaction of biologically active molecules with phospholipid membranes. I. Fluorescence depolarization studies on the effect of polymeric biocide bearing biguanide groups in the main chain. Biochim Biophys Acta.

[31] Gilbert P, Moore LE (2005). Cationic antiseptics: diversity of action under a common epithet. J Appl Microbiol.

[32] Zaki AM, Troisi A, Carbone P (2016). Unexpected like-charge self-assembly of a Biguanide-based antimicrobial polyelectrolyte. J Phys Chem Lett.

[33] Zorko M, Langel Ü (2005). Cell-penetrating peptides: mechanism and kinetics of cargo delivery. Adv Drug Deliv Rev.

[34] Creppy EE, Diallo A, Moukha S, Eklu-Gadegbeku C, Cros D (2014). Study of epigenetic properties of poly (HexaMethylene Biguanide) hydrochloride (PHMB). Int J Environ Res Public Health.

[35] Chindera K, Mahato M, Kumar Sharma A (2016). The antimicrobial polymer PHMB enters cells and selectively condenses bacterial chromosomes. Sci Rep.

[36] Purdue PE, Koulouvaris P, Potter HG, Nestor BJ, Sculco TP (2007). The cellular and molecular biology of periprosthetic osteolysis. Clin Orthop Relat Res.

[37] Revell PA (2008). The combined role of wear particles, macrophages and lymphocytes in the loosening of total joint prostheses. J R Soc Interface.

[38] Mavrogenis AF, Dimitriou R, Parvizi J, Babis GC (2009). Biology of implant osseointegration. J Musculoskelet Neuronal Interact.

[39] Landgraeber S, Jäger M, Jacobs JJ, Hallab NJ (2014). The pathology of orthopedic implant failure is mediated by innate immune system cytokines. Mediat Inflamm.

[40] Mori G, D'Amelio P, Faccio R, Brunetti G (2013). The interplay between the bone and the immune system. Clin Dev Immunol.

[41] Wu AC, Raggatt LJ, Alexander KA, Pettit AR (2013). Unraveling macrophage contributions to bone repair. Bonekey Rep.

[42] Gordon S, Martinez FO (2010). Alternative activation of macrophages, mechanism and functions. Immunity.

[43] van Ginderachter JA, Movahedi K, Hassanzadeh Ghassabeh G (2011). Classical and alternative activation of mononuclear phagocytes: picking the best of both worlds for tumor promotion. Immunobiology.

[44] Mills CD, Thomas AC, Lenz LL, Munder M (2014). Macrophage, SHIP of Immunity. Front Immunol.

[45] Graney PL, Roohani-Esfahani S-I, Zreiqat H, Spiller KL (2016). In vitro response of macrophages to ceramic scaffolds used for bone regeneration. J R Soc Interface.

[46] Galli SJ, Borregaard N, Wynn TA (2011). Phenotypic and functional plasticity of cells of innate immunity, macrophages, mast cells and neutrophils. Nat Immunol.

[47] Gratchev A, Kzhyshkowska J, Köthe K (2006). Mφ1 and Mφ2 can be re-polarized by Th2 or Th1 cytokines, respectively, and respond to exogenous danger signals. Immunobiology.

[48] Riabov V, Salazar F, Htwe SS (2017). Generation of anti-inflammatory macrophages for implants and regenerative medicine using self-standing release systems with a phenotype-fixing cytokine cocktail formulation. Acta Biomater.

[49] Sridharan R, Cameron AR, Kelly DJ, Kearney CJ, O’Brien FJ (2015). Biomaterial based modulation of macrophage polarization, a review and suggested design principles. Mater Today.

[50] Hotchkiss KM, Ayad NB, Hyzy SL, Boyan BD, Olivares-Navarrete R (2017). Dental implant surface chemistry and energy alter macrophage activation in vitro. Clin Oral Implants Res.

[51] Hotchkiss KM, Reddy GB, Hyzy SL, Schwartz Z, Boyan BD, Olivares-Navarrete R (2016). Titanium surface characteristics, including topography and wettability, alter macrophage activation. Acta Biomater.

[52] Biswas SK, Mantovani A (2010). Macrophage plasticity and interaction with lymphocyte subsets, cancer as a paradigm. Nat Immunol.

[53] Ruytinx P, Proost P, van Damme J, Struyf S (2018). Chemokine-induced macrophage polarization in inflammatory conditions. Front Immunol.

[54] Shapouri-Moghaddam A, Mohammadian S, Vazini H (2018). Macrophage plasticity, polarization, and function in health and disease. J Cell Physiol.

[55] Tsuchiya S, Yamabe M, Yamaguchi Y, Kobayashi Y, Konno T, Tada K (1980). Establishment and characterization of a human acute monocytic leukemia cell line (THP-1). Int J Cancer.

[56] Auwerx J (1991). The human leukemia cell line, THP-1: a multifacetted model for the study of monocyte-macrophage differentiation. Experientia.

[57] Sousa-Vasconcelos PS, Seguins WS, Luz ES, de Pinho RT (2015). Pattern of cytokine and chemokine production by THP-1 derived macrophages in response to live or heat-killed Mycobacterium bovis bacillus Calmette-Guérin Moreau strain. Mem Inst Oswaldo Cruz.

[58] Kamendulis LM (2008). Studies to elucidate the potential involvement of the Kupffer cell in PHMB mouse liver hemangiosacromas.

[59] Mittal M, Siddiqui MR, Tran K, Reddy SP, Malik AB (2014). Reactive oxygen species in inflammation and tissue injury. Antioxid Redox Signal.

[60] Kim HR, Shin DY, Chung KH (2017). In vitro inflammatory effects of polyhexamethylene biguanide through NF-κB activation in A549 cells. Toxicol in Vitro.

[61] Rao AJ, Gibon E, Ma T, Yao Z, Smith RL, Goodman SB (2012). Revision joint replacement, wear particles, and macrophage polarization. Acta Biomater.

[62] Ishimi Y, Miyaura C, Jin CH (1990). IL-6 is produced by osteoblasts and induces bone resorption. J Immunol.

[63] Gu Q, Yang H, Shi Q (2017). Macrophages and bone inflammation. J Orthop Transl.

[64] Ainslie KM, Tao SL, Popat KC (2009). In vitro inflammatory response of nanostructured titania, silicon oxide, and polycaprolactone. J Biomed Mater Res A.

[65] Barth KA, Waterfield JD, Brunette DM (2013). The effect of surface roughness on RAW 264.7 macrophage phenotype. J Biomed Mater Res A.

[66] Refai AK, Textor M, Brunette DM, Waterfield JD (2004). Effect of titanium surface topography on macrophage activation and secretion of proinflammatory cytokines and chemokines. J Biomed Mater Res A.

[67] Filova E, Brynda E, Riedel T (2014). Improved adhesion and differentiation of endothelial cells on surface-attached fibrin structures containing extracellular matrix proteins. J Biomed Mater Res A.

[68] Kieswetter K, Schwartz Z, Dean DD, Boyan BD (1996). The role of implant surface characteristics in the healing of bone. Crit Rev Oral Biol Med.

[69] Sunarso TR, Tsuru K, Ishikawa K (2016). A superhydrophilic titanium implant functionalized by ozone gas modulates bone marrow cell and macrophage responses. J Mater Sci Mater Med.

[70] Amarasekara DS, Yun H, Kim S, Lee N, Kim H, Rho J (2018). Regulation of osteoclast differentiation by cytokine networks. Immune Netw.

[71] Liu X, Chen Z, Lan T, Liang P, Tao Q (2019). Upregulation of interleukin-8 and activin a induces osteoclastogenesis in ameloblastoma. Int J Mol Med.

[72] Lencel P, Delplace S, Hardouin P, Magne D (2011). TNF-alpha stimulates alkaline phosphatase and mineralization through PPARgamma inhibition in human osteoblasts. Bone.

