# Supplementary figures and images for: In vitro response of THP-1 derived macrophages to antimicrobially effective PHMB-coated Ti6Al4V alloy implant material with and without contamination with S. epidermidis and P. aeruginosa
Source: Biomater Res. 2022 Jan 9;26:1. doi: 10.1186/s40824-021-00247-1 (PMC8744236; doi:10.1186/s40824-021-00247-1)

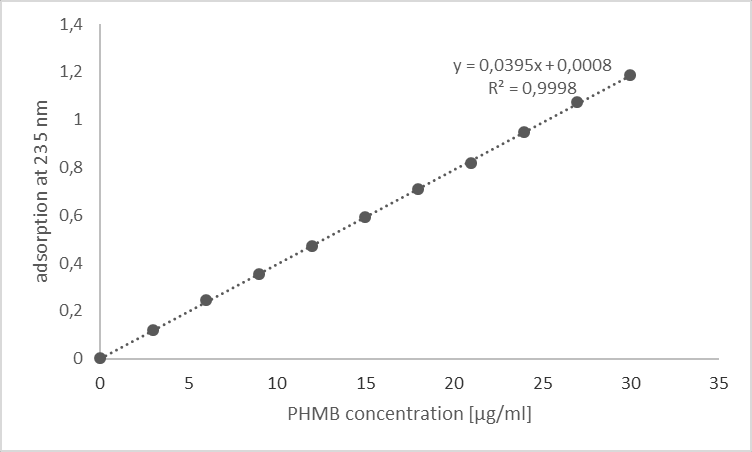

Supplement: Supplementary file 1 — Additional file 1: Supplementary Fig. 1. Calibration curve of PHMB in water. Two hundred fifty microlitre of aqueous PHMB solution with rising concentration were added to each well of a 96 well quartz plate and adsorption was measured at 235 nm. N = 4. [file 40824_2021_247_MOESM1_ESM.tif]
